# Supplementary material for: Increasing trend of scientists to switch between topics
Source: Nat Commun. 2019 Jul 31;10:3439. doi: 10.1038/s41467-019-11401-8 (PMC6668429; doi:10.1038/s41467-019-11401-8)
Supplement: Supplementary file 1 — Supplementary Information [file 41467_2019_11401_MOESM1_ESM.pdf]

Supplementary Information

Increasing trend of scientists to switch between topics

An Zeng, Zhesi Shen, Jianlin Zhou, Ying Fan, Zengru Di,
Yougui Wang, H. Eugene Stanley, and Shlomo Havlin

Supplementary figures

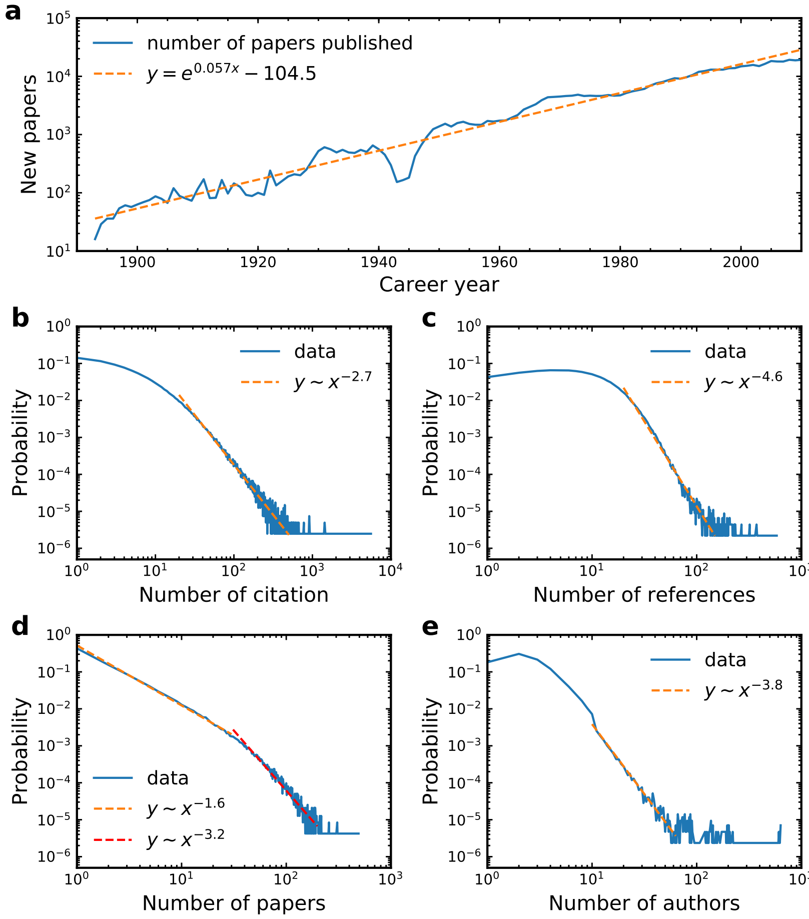

Supplementary Figure 1. Basic statistic of the APS data. (a) Exponential fit to the yearly growth of new papers in the APS data set, with the trend consistent with that in the literature [1]. (b) Power-law fit to papers' citation distribution. (c) Power-law fit to papers' number of reference distribution. (d) Two power-law fits to authors' productivity (i.e. number of published papers of an author) distribution, in different regimes. (e) Power-law fit to papers' team size (i.e. number of authors) distribution. The fat long tail in (e) is due to papers from experimental nuclear and particle physics that have a huge number of authors.

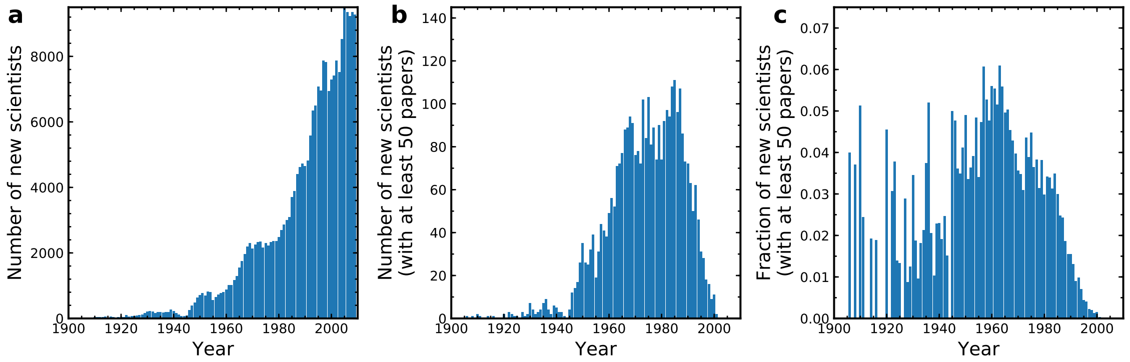

Supplementary Figure 2. Number of new scientists in different years. (a) The yearly number of new authors in APS data set. (b) The yearly number of new authors who will have at least 50 papers in APS data set. (c) The yearly fraction of new authors who will have at least 50 papers in APS data set. This fraction is generally stable over time, with a decreasing trend after 1990. This is because our data set ends in 2010 and the scientists who started their career after 1990 do not have enough time in the data set to cumulate at least 50 papers.

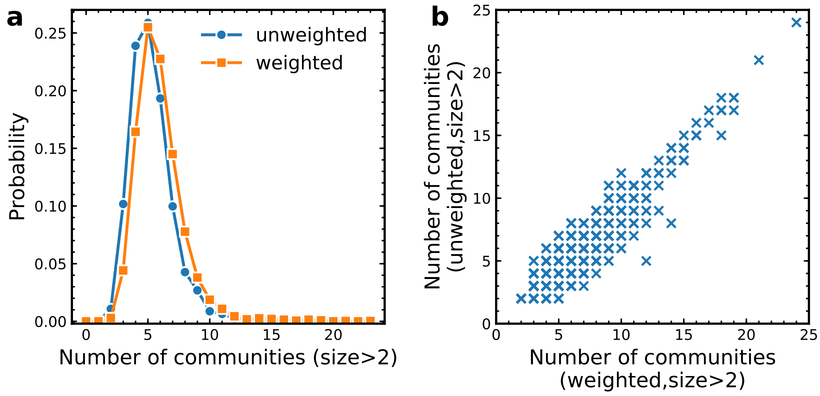

Supplementary Figure 3. Comparison of the weighted and unweighted co-citing networks. (a) The distributions of the number of communities in scientists' weighted and unweighted co-citing networks. Small clusters with no more than 2 nodes are filtered. In the weighted co-citing networks, the weight of a link is the number of common references between two connected papers. The communities in the weighted co-citing networks are obtained by maximizing the weighted modularity function [2]. (b) The scatter plot of the number of communities in scientists' weighted co-citing networks and the number of communities in their unweighted co-citing networks. The community structure is not significantly altered by considering weights, as links within communities tend to have large weights.

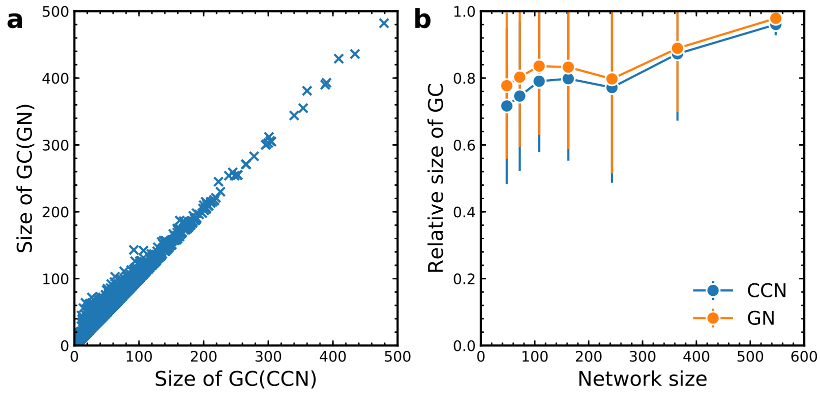

Supplementary Figure 4. Networks with both co-citing and co-cited links. (a) For each scientist, we construct a co-citing network (CCN) based on the co-citing relations between her papers. We can also construct an aggregated network (GN) where any pair of her papers are linked if they either co-cite at least one reference or are co-cited by at least one paper. We compare the difference between CCN and GN by making a scatter plot of the size of the giant component in these two networks. Most of the points are located very near the diagonal, indicating that using co-citing relations can well capture the overall relations between papers. (b) The relative sizes of giant component (GC) of CCN and GN for scientists with different number of papers, showing that the GCs are generally a large fraction of the network for all scientists in both CCN and GN. The error bars here represent standard deviations.

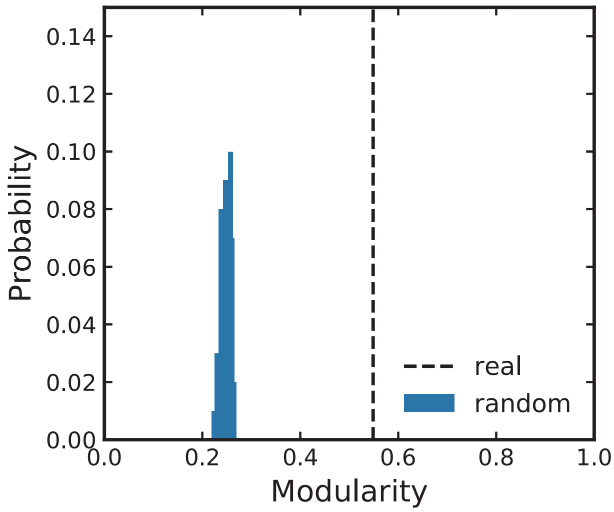

Supplementary Figure 5. Illustration of the significant difference between Q_{real} and Q_{rand}. The modularity Q_{real} of the co-citing network of a typical scientist compared to the distribution of the modularity Q_{rand} of its 100 degree-preserved reshuffled counterparts.

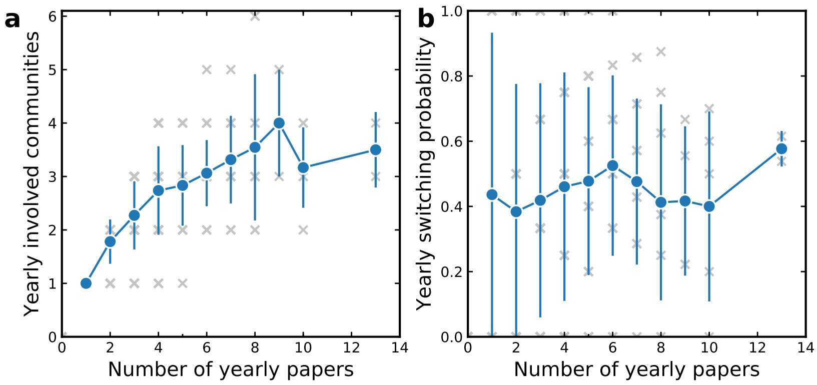

Supplementary Figure 6. Dependence of yearly involved communities and switching probability on yearly publications. (a) The yearly involved communities versus the yearly published papers for all scientists in each of their first 40 career years. When a scientist publishes more papers in a year, he/she will have higher number of yearly involved communities purely by chance. (b) The yearly switching probability versus the yearly published papers for all scientists in each of their first 40 career years. The error bars in this figure represent standard deviations. The results demonstrate that the yearly switching probability is not correlated with the yearly published papers, and thus can be used as an unbiased metric for quantifying scientists' switching behavior between communities.

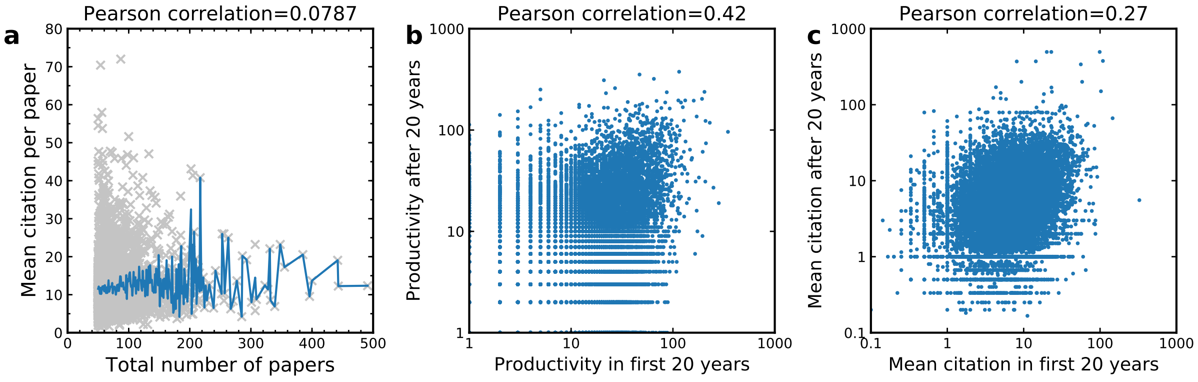

Supplementary Figure 7. Properties of two metrics measuring research performance. (a) Scatter plot of the number of published papers and the mean citation per paper of each scientist (Pearson correlation is 0.0787). The blue curve shows the average trend, indicating that these two performance metrics are almost uncorrelated. (b) Scatter plot of the productivity (i.e. number of published papers) in scientists' first 20 career years and after scientists' 20 career years (Pearson correlation is 0.42). (c) Scatter plot of the mean citation per paper in scientists' first 20 career years and after scientists' 20 career years (Pearson correlation is 0.27). The results suggest that the top productive scientists we considered in the manuscript are in general productive in both stages of their careers. Similarly, the top scientists with highest mean citation per paper tend to have high citation per paper in each stage of their careers.

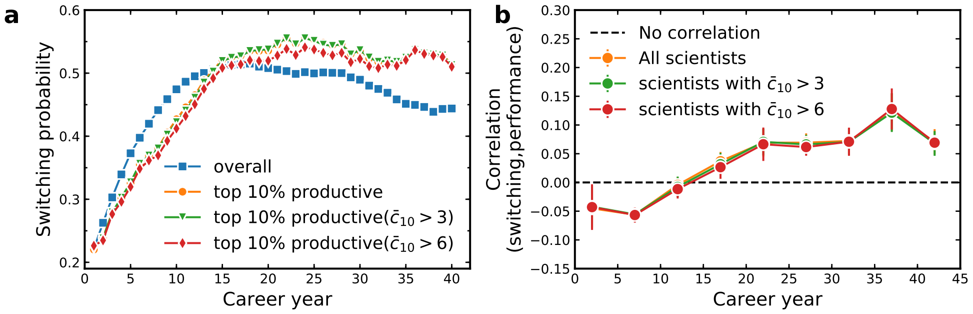

Supplementary Figure 8. Excluding productive scientists with low citations. (a) Comparison of the overall switching probability (all scientists) with the switching probability of the 10% most productive scientists in different career years. The switching probability of the top 10% productive scientists with mean citations $\bar{c}_{10}$ higher than 3 and 6 are also presented for comparison. (b) The Pearson correlation between scientists' switching probability in different career years and their productivity. The correlation is also measured when the scientists with low mean citations ($\bar{c}_{10} \leq 3$ or $\bar{c}_{10} \leq 6$) are excluded. The error bars here represent standard deviations.

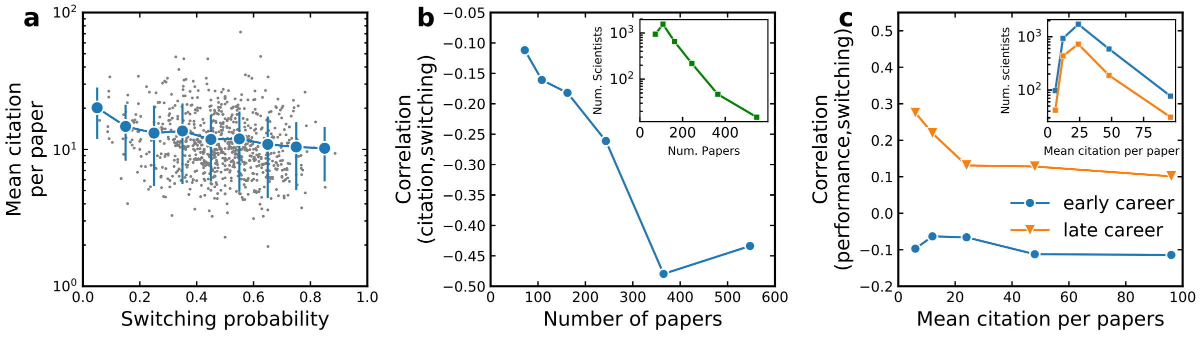

Supplementary Figure 9. Correlation between switching probability and research performance. (a) The mean citation per paper versus switching probability for scientists who published between 70 and 90 papers in their career. The error bars here represent standard deviations. The downward trend shown by the averaged curve indicates that the switching probability and mean citation per paper is slightly negatively correlated. (b) The Pearson correlation between the switching probability and mean citations per paper for scientists of different productivity (number of papers in their career) ranges. One can see that the correlation between switching probability and mean citation per paper is negatively correlated for each group of scientists, and the negative correlation is more significant for more productive scientists. (c) The Pearson correlation between productivity and mean switching probability. The results of early career correlation ($< 5y$) and later career correlation ($> 30y$) are presented. The correlations between productivity and switching probability are stronger for the scientists with relatively low mean citations (below 20 but above 5). The insets in this figure are the number of scientists in each bin for calculating the Pearson correlation coefficient.

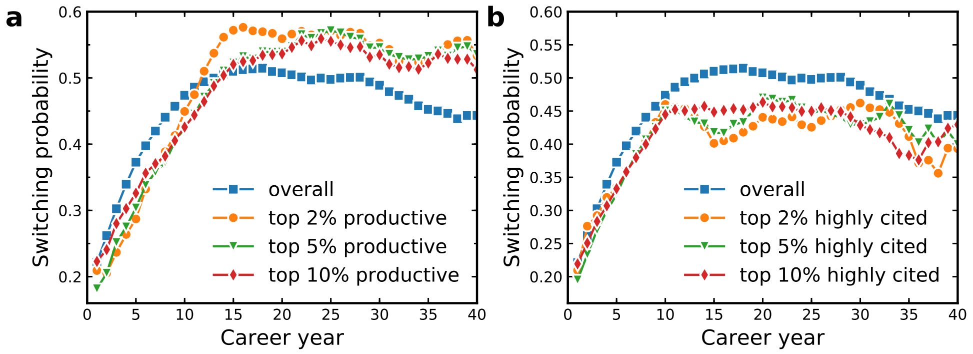

Supplementary Figure 10. Switching probability of top performing scientists. (a) Comparison of the overall switching probability (all scientists) with the switching probability of the 2%, or 5% or 10% most productive scientists in different career years. (b) Comparison of the overall switching probability (all scientists) with the switching probability of the 2%, or 5% or 10% scientists who has the highest mean citation c_{10} per paper.

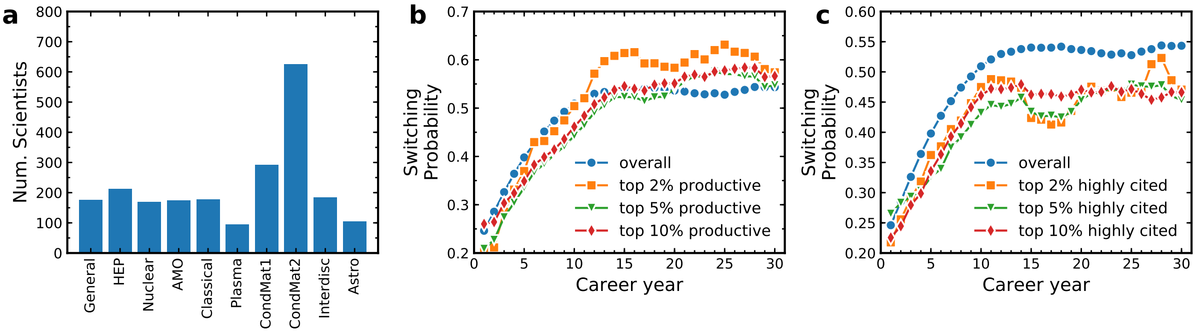

Supplementary Figure 11. Controlling topic areas when selecting top performing scientists. (a) The distribution of the scientists in each subfield. We use PACS codes to identify the general topic area of scientists. The PACS classification uses four digits and an extra identifier. The first digit identifies 10 different physics subfields. As PACS codes are only enforced in APS journals from 1985 to 2015, a large number of papers do not have such codes. Among the scientists with at least 50 papers, we select those who have at least 70% papers with PACS codes, resulting in 2210 scientists. In order to control topic areas when computing the percentiles of best performing scientists, we assign each scientist to only one subfield (according to the first digit of the PACS code). Scientists may have papers belonging to multiple subfields, but some of these might not be significant. Here, we use the Revealed Comparative Advantage (RCA) index [3] to assign each scientist only to the subfield on which their engagement is most significant. Here, $RCA_{i\alpha} = \frac{w_{i\alpha}/\sum_{\beta}(w_{i\beta})}{\sum_{j} w_{j\alpha}/\sum_{j\beta} w_{j\beta}}$ where $w_{i\alpha}$ is an integer corresponding to the number of publications of author i in subfield α. Each scientist is assigned to a subfield in which he/she has the highest RCA value. The results in (a) shows that the scientists with at least 50 papers are from different subfields. (b) Comparison of the overall switching probability (all scientists) with the switching probability of the 2%, or 5% or 10% most productive scientists in different career years. (c) Comparison of the overall switching probability (all scientists) with the switching probability of the 2%, or 5% or 10% scientists who has the highest mean citation per paper. In (b) and (c), the topic areas are controlled when computing the percentiles. Specifically, in (b) we take the top 10% (or 2% or 5%) most productive scientists from each subfield, forming the top performing scientists. In (c) we take the top 10% (or 2%, or 5%) scientists in each subfield whose publications has highest mean c_{10}, forming the top performing scientists.

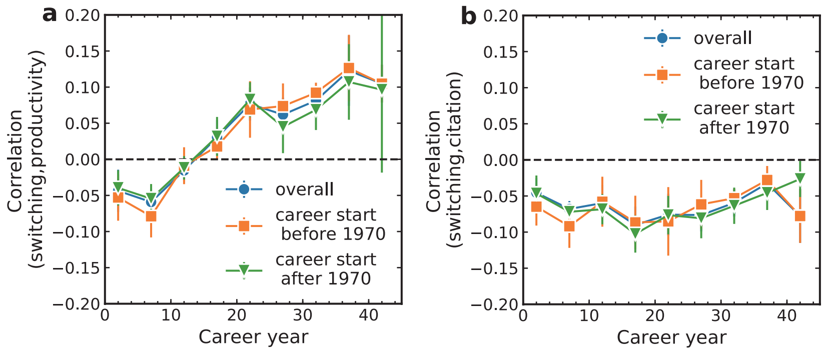

Supplementary Figure 12. Correlation between switching probability and research performance in different career stages. (a) The Pearson correlation between scientists' switching probability in different career years and their overall productivity. We find that high switching probability in early career is correlated to low overall productivity (negative correlations), while high switching probability in later career is associated with high overall productivity (positive correlations). (b) The Pearson correlation between scientists' switching probability in different career years and the mean citations per paper. The average citation per paper is negatively correlated with the switching probability in all career periods. The correlations are robust for scientists who start their careers in different years. The error bars in this figure represent standard deviations.

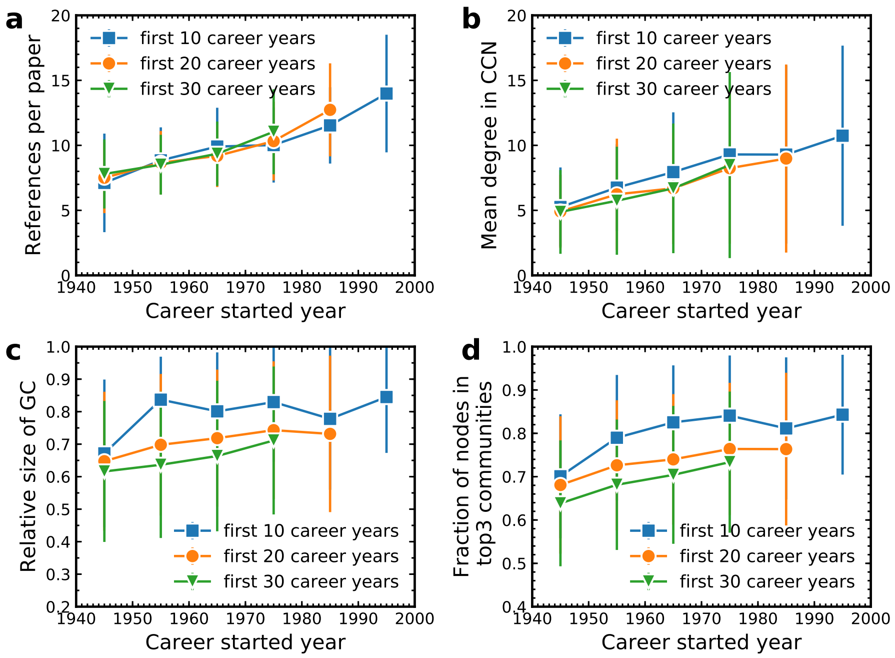

Supplementary Figure 13. Evolution of structural properties of the co-citing networks (CCNs). (a) The mean number of references per paper published by scientists who started their career in different years. (b) The mean degree of the CCNs of scientists who started their career in different years. (c) The mean relative sizes of GC of the CCNs of scientists who started their career in different years. (d) The mean fraction of nodes in the 3 largest communities in the CCNs of scientists who started their career in different years. The error bars in this figure represent standard deviations.

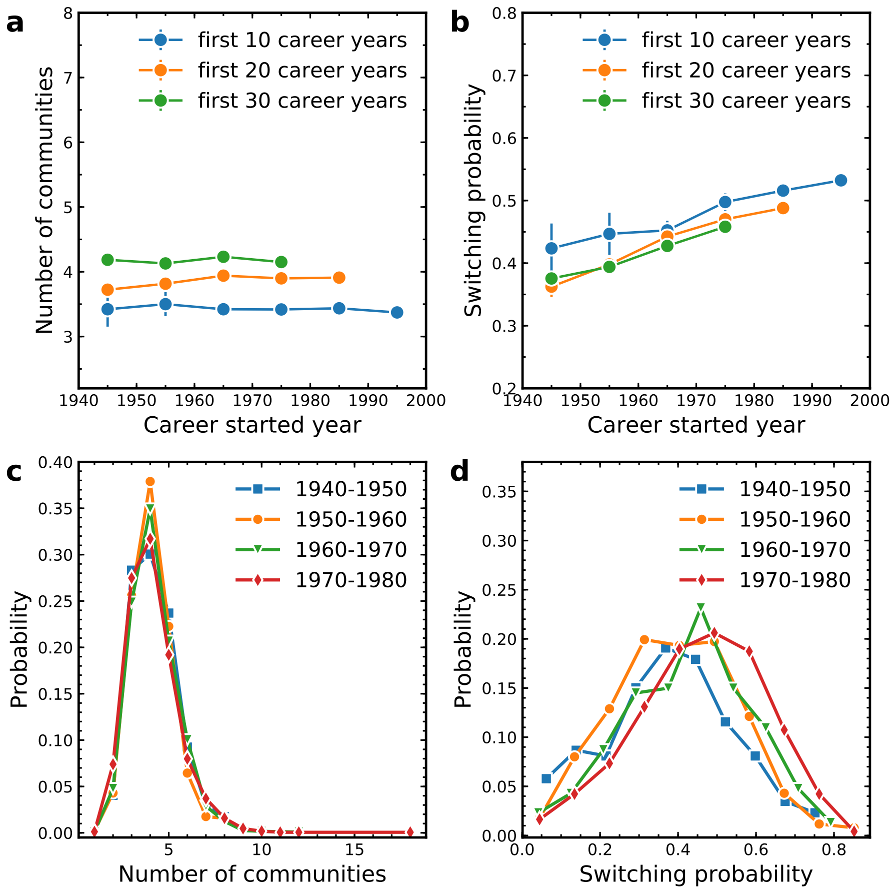

Supplementary Figure 14. Switching probability with standard error of the mean in different years. (a) The mean number of communities of scientists who started their career in different years. (b) The average switching probability of scientists who started their career in different years. The error bars are the standard error of the mean. (c) Distributions of the number of communities for scientists who started their career between 1940 and 1950, for those between 1950 and 1960, for those between 1960 and 1970, and for those between 1970 and 1980. (d) Distributions of the switching probability for scientists who started their career between 1940 and 1950, for those between 1950 and 1960, for those between 1960 and 1970, and for those between 1970 and 1980.

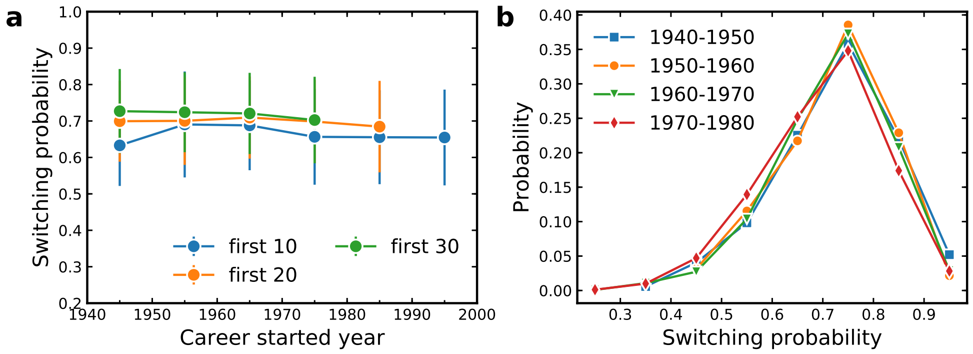

Supplementary Figure 15. Switching probability of scientists in a null model. (a) The average switching probability of scientists in the null model who started their career in different years. For each scientist, we only consider the first y career years to obtain a fairer comparison of scientists with different career length. The error bars here represent standard deviations. (b) Distributions of the switching probability for scientists in the null model who started their career between 1940 and 1950, for those between 1950 and 1960, for those between 1960 and 1970, and for those between 1970 and 1980. The null model is used to remove the effect of increasing number of papers and scientists. In the null model, we preserve the published papers for each scientist, yet we reshuffled the time order of these papers. Thus, the detected communities in each scientist's co-citing network is kept unchanged while the switching probability over his/her career will be altered. The switching probability in this null model is stable over the years, different from the increasing trend observed in the real data. In this null model, the number of papers and scientists grow exponentially the same as for the real data. Therefore, the results suggest that the increasing trend of switching probability in real data is not caused by the increasing number of papers and scientists.

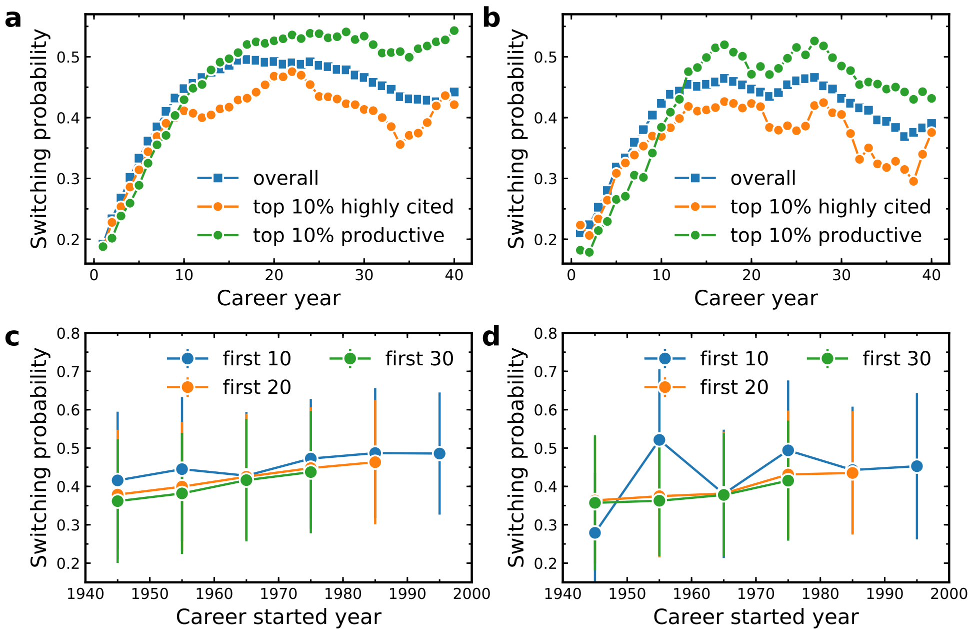

Supplementary Figure 16. Impact of collaborative effects on the findings. (a)(b) Comparison of the overall switching probability (all scientists) with the switching probability of the 10% most influential scientists in different career years. The influence of scientists is respectively measured as number of publications and citations per paper. (c)(d) The average switching probability of scientists who started their career in different years. For each scientist, we only consider the first y career years for a fairer comparison of scientists with different career length. In this figure, in the case of multi-authored papers we assign a paper credit among authors using the collective credit allocation approach [4]. This method assigns credits based on the community perception, i.e., each citing paper expresses its perception of the scientific impact of a paper's coauthors by citing other papers published by the same authors on the same subject. We thus filter out a scientist's papers in which the credit share of the scientist is lower than a certain value ϵ ($\epsilon = 0.2$ in (a)(c), $\epsilon = 0.4$ in (b)(d)). After filtering out these papers, we re-analyze the individual and collective switching patterns of scientists. Although the results are noisier due to the smaller sample size after data filtering, there is no qualitative difference compared to our previous results presented in the manuscript (Figs. 3cd and 4ab), suggesting that our findings are robust to co-authorship effects. The error bars in this figure represent standard deviations.

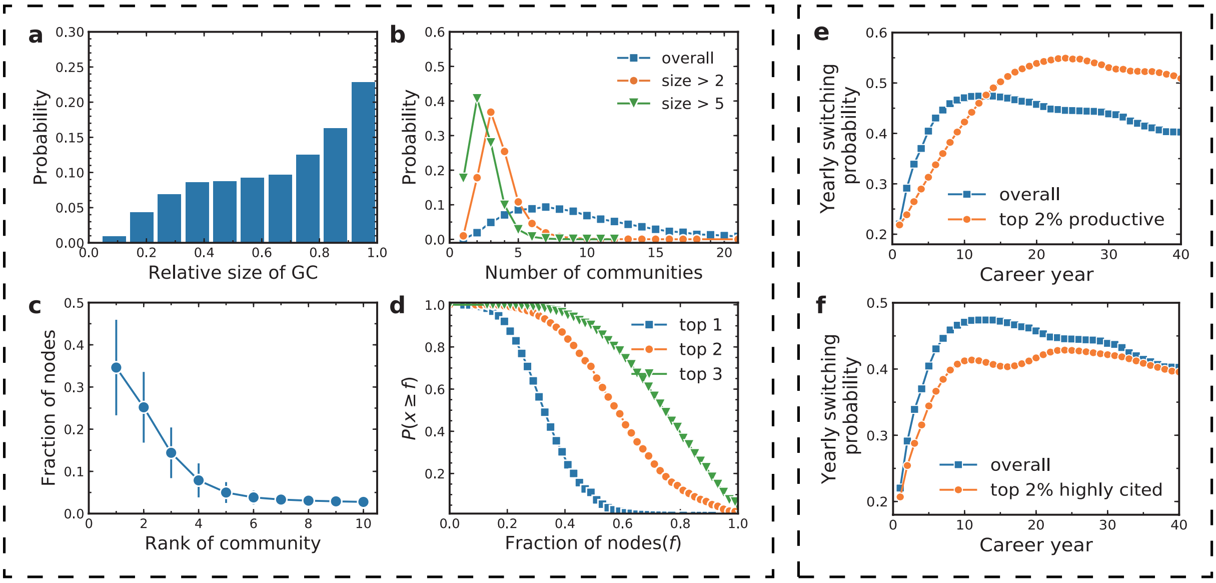

Supplementary Figure 17. The structural and switching dynamics analysis of scientists with at least 20 papers. In the paper, we only consider scientists with at least 50 papers in order to ensure that the network is sufficiently large to obtain meaningful community detection results. Here, we test an alternative case where all the scientists with at least 20 papers are considered, which results in 15373 scientists in our data set. This figure shows the structural analysis results. (a) The distribution of the relative size of GC. (b) The distribution of the number of communities for all scientists. The three curves respectively represent the cases where all communities are preserved, small communities with fewer than 2 nodes are eliminated, and small communities with fewer than 5 nodes are eliminated. (c) Fraction of papers in different communities sorted by descending size. The error bars here represent standard deviations. (d) Inverse cumulative probability of fraction of nodes in the biggest community (legend as top 1), the two largest communities (legend as top 2), and the three largest communities (legend as top 3), respectively. (e) Comparison of the overall switching probability with the switching probability of the 2% most productive scientists in different career years. (f) Comparison of the overall switching probability with the switching probability of the 2% scientists who has the highest mean citation per paper.

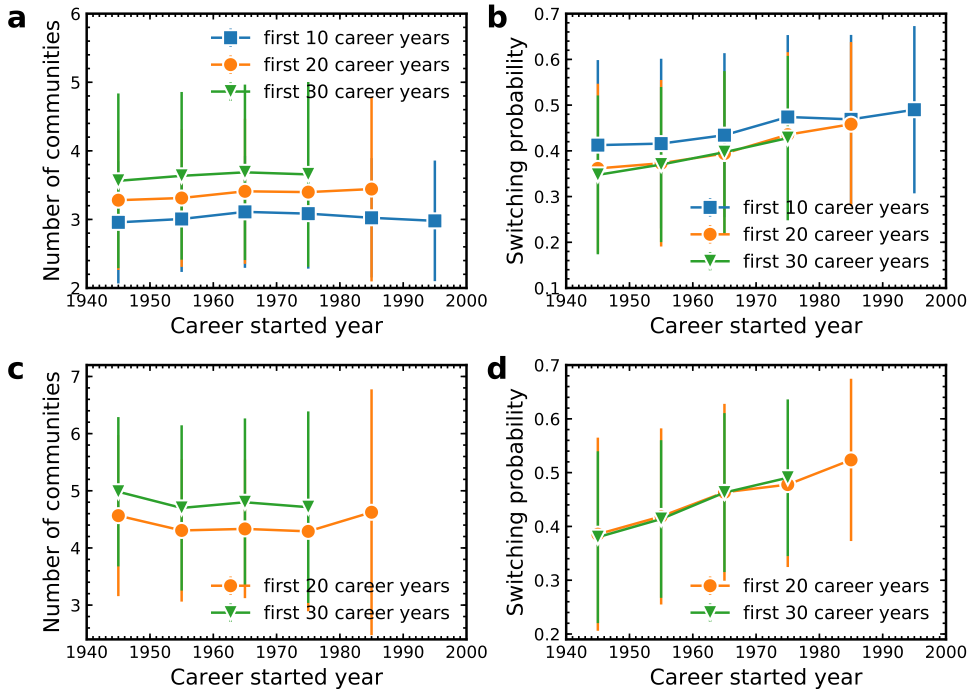

Supplementary Figure 18. Evolution of community number and switching probability for scientists with at least 20 or 50 papers. When we study the evolution of the structural and dynamical properties of CCNs as the development of science, we only consider scientists with at least 30 papers in their first y career years ($y = 10, 20, 30$) in order to ensure that the network is sufficiently large to obtain meaningful community detection results. Here, we test two alternative cases where we require the scientists to have at least 20 (see Fig. S18ab) and 50 (See Fig. S18cd) papers in their first y career years. (a)(c) The mean number of communities for scientists who started their career in different years. (b)(d) The average switching probability of scientists who started their career in different years. The results for $y = 10$ are not included in (c) and (d) as there are very few scientists who published over 50 papers in their first 10 career years. The error bars in this figure represent standard deviations.

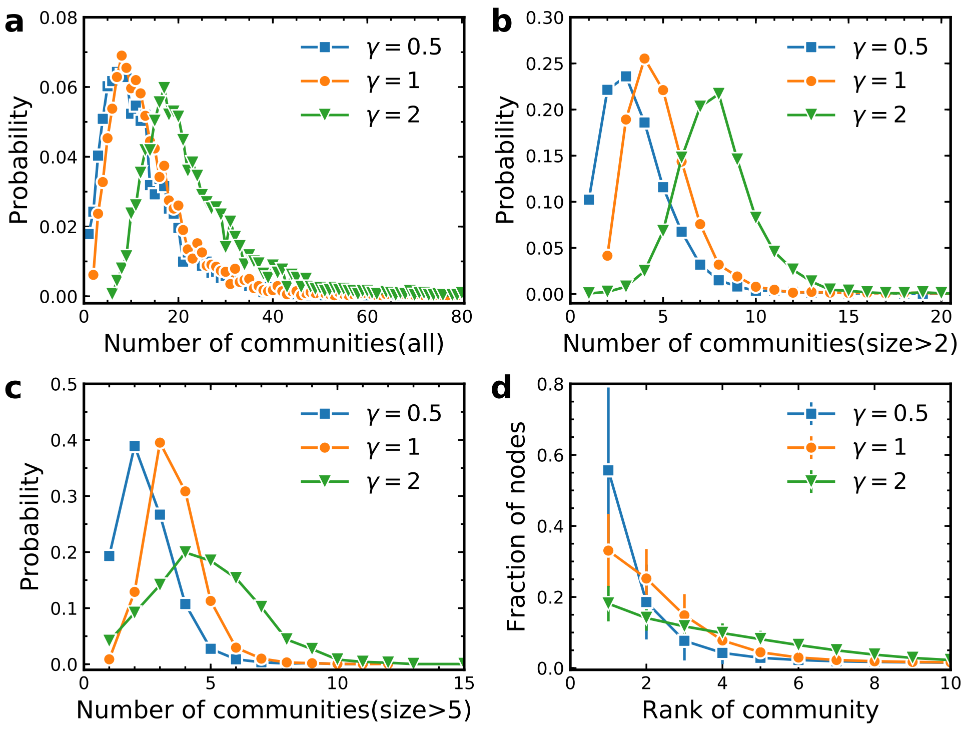

Supplementary Figure 19. Impact of community detection resolutions on the detected communities. In the paper, we perform community detection by maximizing the standard modularity function. Here, we test the results with the modified modularity function with a resolution parameter γ where a larger γ yields more communities. Apart from the standard $\gamma = 1$, we consider two typical values of γ (i.e. $\gamma = 0.5$ and $\gamma = 2$) as suggested in ref. [5]. (a) The distributions of the number of communities for all scientists under different resolution parameter γ. (b) The distribution of the number of communities (size>2) for all scientists under different resolution parameter γ. (c) The distribution of the number of communities (size>5) for all scientists under different resolution parameter γ. Although the number of communities is influenced by the parameter γ, the distributions after filtering out small clusters (e.g. size>2 and size>5) are still narrow. (d) Fraction of nodes in different communities under different parameter γ. The error bars here represent standard deviations.

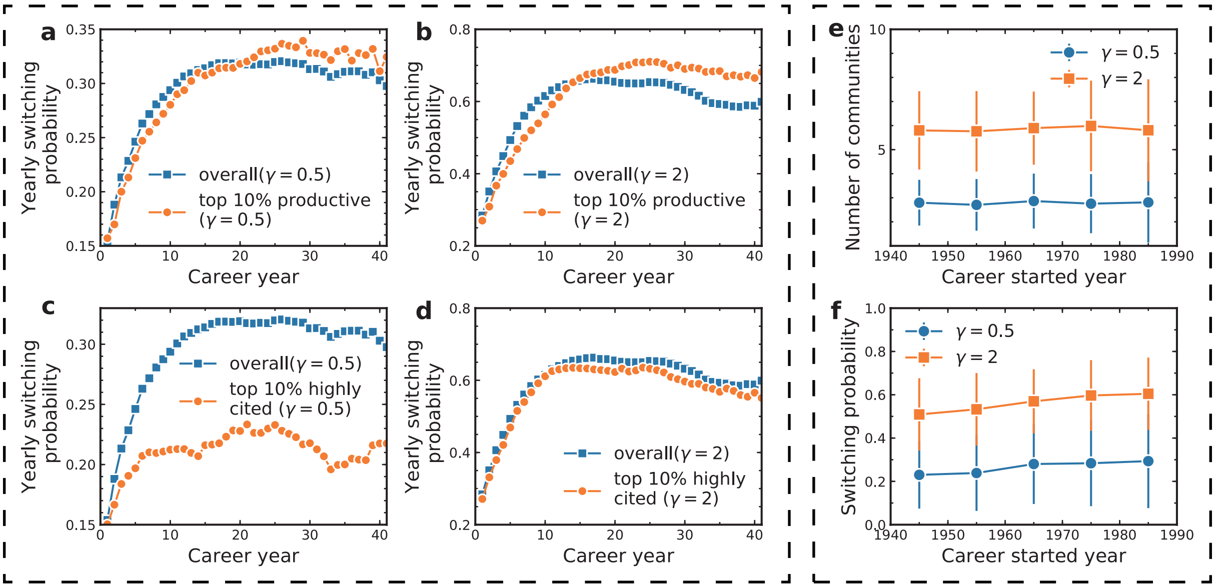

Supplementary Figure 20. Impact of community detection resolutions on the evolution of switching probability. In the paper, we perform community detection by maximizing the standard modularity function. Here, we test the results with the modified modularity function with a resolution parameter γ where a larger γ yields small but more communities, and vice versa. (a)(b) Comparison of the overall switching probability with the switching probability of the 10% most productive scientists in different career years. (c)(d) Comparison of the overall switching probability with the switching probability of the 10% scientists who has the highest mean citation per paper. $\gamma = 0.5$ in (a)(c), while $\gamma = 2$ in (b)(d). The large gap in (c) suggests that frequent switching between very dissimilar topics may cause significantly adverse effect on mean citation per paper. (e) The mean number of communities for scientists who started their career in different years (showing both $\gamma = 0.5$ and $\gamma = 2$ cases). (f) The average switching probability of scientists who started their career in different years (showing both $\gamma = 0.5$ and $\gamma = 2$ cases). In (e)(f), we only consider scientists' first 20 career years when we compare scientists who started their careers in different years. The error bars in this figure represent standard deviations.

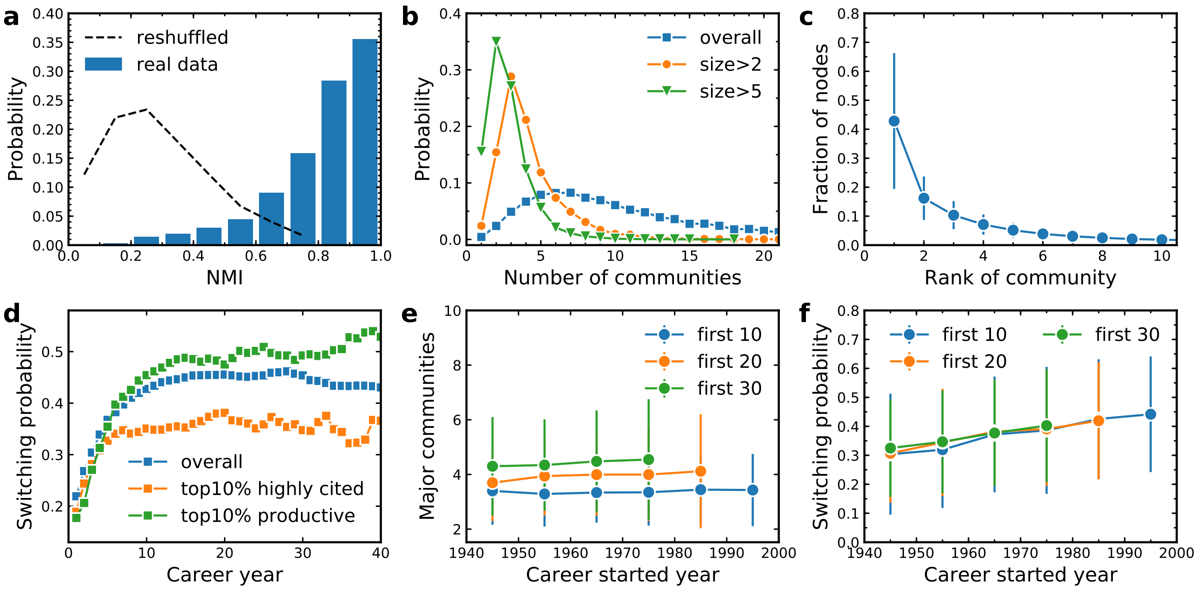

Supplementary Figure 21. The figure summarizes the results based on the Infomap method [6]. Infomap is a community detection algorithm which is independent of modularity maximization. It is found that the resolution limit is orders of magnitudes smaller for Infomap compared to modularity [7]. (a) The distribution of NMI between the communities detected based Infomap and modularity. For comparison, we reshuffled the nodes between the communities detected by infomap, and present also the distribution of NMI between the reshuffled communities and the communities detected by modularity maximization. (b) The distribution of the number of communities for all scientists. Small communities with less than 3 nodes are eliminated (legend as size>2), and small communities with less than 6 nodes are eliminated (legend as size>5). (c) Fraction of papers in different communities. (d) The switching probability of scientists in different career years. The switching probability of the 10% most productive scientists and 10% scientists who have the highest mean citation per paper are shown for comparison. (e) The mean number of communities (size>2) of scientists who started their career in different years. (f) The average switching probability of scientists who started their career in different years. For fair comparison of scientists from different years, we only consider here scientists' first y career years. Here, $y = 10$, 20 and 30. The error bars in this figure represent standard deviations.

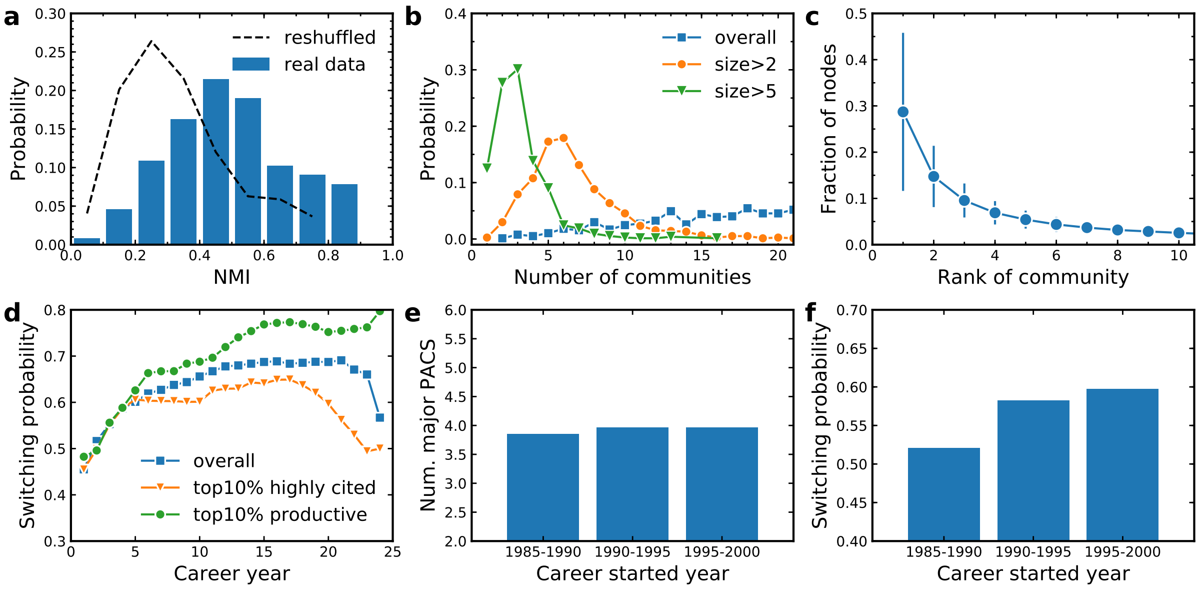

Supplementary Figure 22. The figure summarizes the results based on the PACS codes. For a scientist, if two of his/her papers share the same first 4 digits of the primary PACS codes (i.e. the first PACS code in a paper), we consider these two papers to belong to the same topic. We consider the scientists who published their first paper in APS after 1985 (as PACS codes are enforced by APS only from 1985 to 2015). (a) The distribution of NMI between the topics detected based PACS codes and modularity method. For comparison, we show also the distribution of NMI between the topics detected based reshuffled PACS codes and modularity method. (b) The distribution of the number of topics for all scientists. Small topics with less than 3 papers are eliminated (legend as size>2), and small topics with less than 6 papers are eliminated (legend as size>5). (c) Fraction of papers in different topics. The error bars here represent standard deviations. (d) The switching probability of scientists in different career years. The switching probability of the 10% most productive scientists and 10% scientists who have the highest mean citation per paper are shown for comparison. (e) The mean number of topics (size>2) of scientists who started their career in different years. The p-value is 0.053 for the Kolmogorov-Smirnov test of the topic number distributions in 1985-1990 and 1995-2000. (f) The average switching probability of scientists who started their career in different years. The p-value is 1.3×10^{-5} for the Kolmogorov-Smirnov test of the switching probability distributions in 1985-1990 and 1995-2000. For fair comparison of scientists from different years, in (e) and (f) we only consider scientists' first 10 career years.

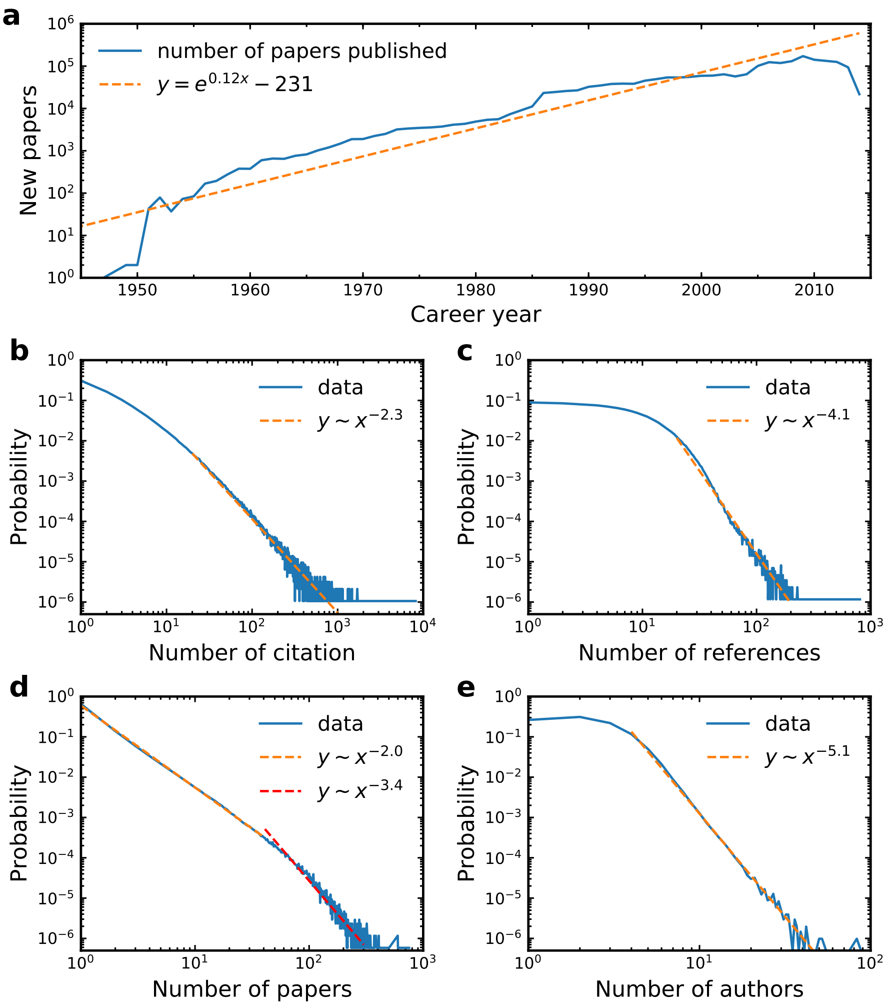

Supplementary Figure 23. Basic statistics for the computer science data. (a) Exponential fit to the growth of yearly new papers in the computer science data set. (b) Power-law fit to papers' citation distribution. (c) Power-law fit to papers' reference distribution. (d) Double power-law fits to authors' productivity (i.e. number of published papers) distribution for different regimes. (e) Power-law fit to papers' team size (i.e. number of authors) distribution.

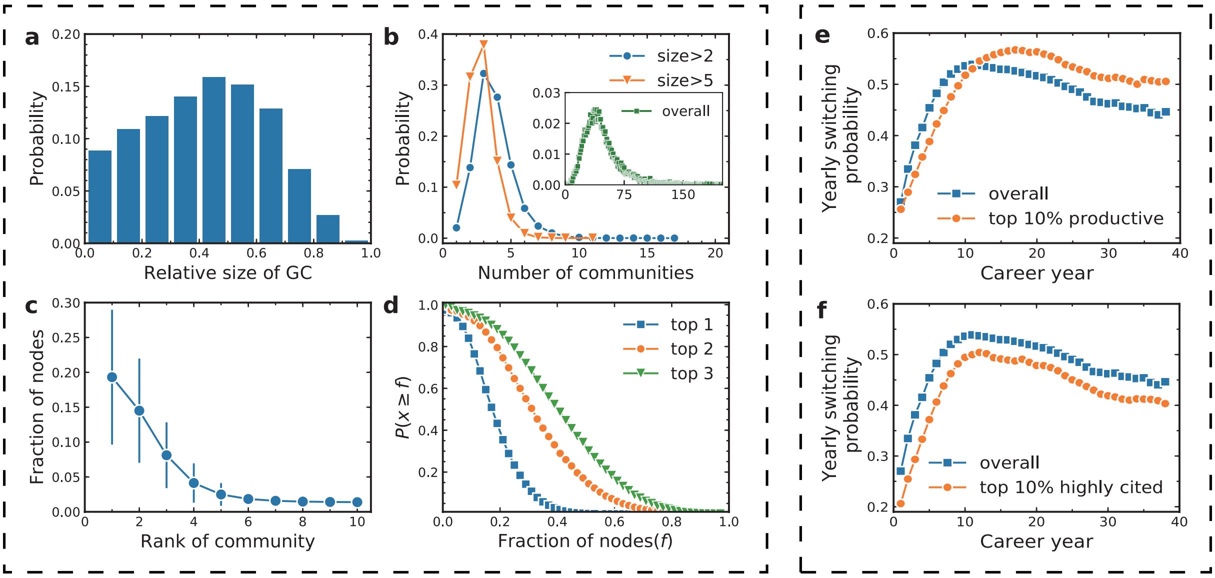

Supplementary Figure 24. The structural and switching dynamics analysis of the computer science data. (a) Distribution of the relative size of giant component (GC). The results suggest that the co-citing networks of individual computer scientists are in general less connected than those of physicists. (b) Distribution of the number of communities for all scientists. The inset shows the distribution of the number of communities without filtering any small communities. (c) Fraction of papers in different communities sorted by descending size. The error bars here represent standard deviations. (d) Inverse cumulative probability of fraction of nodes in the largest community (legend as top 1), the two largest communities (legend as top 2), and the three largest communities (legend as top 3), respectively. (e) Comparison of the overall switching probability with the switching probability of the 10% most productive scientists in different career years. (f) Comparison of the overall switching probability with the switching probability of the 10% scientists who has the highest mean citation per paper.

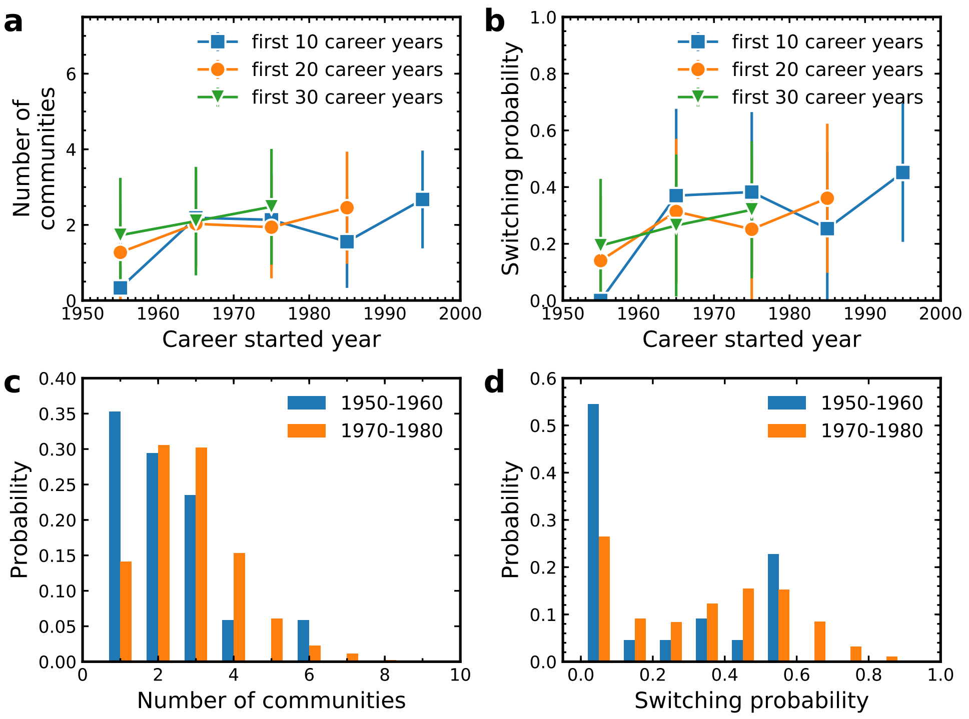

Supplementary Figure 25. The evolution of the structural and dynamical properties of CCNs as the development of computer science. Similar to the APS data, we only consider scientists' first y career years and remove (i) all the scientists who not yet reached y years career, and (ii) those who published less than 30 papers in their first y career years. The results for $y = 10, 20, 30$ are presented respectively in this figure. (a) The mean number of communities for scientists who started their career in different years. The increasing trend is because the co-citing networks of computer scientists are in general very sparse and thus have many isolated nodes. Papers become more connected in the interdisciplinary era, resulting in the connection of isolated nodes and thus a higher number of clusters with size larger than two. (b) The average switching probability of scientists who started their career in different years. The increasing trend suggests that computer scientists switch between different communities more frequently nowadays. The large fluctuation in $y = 10$ and $y = 20$ is because there are only a small number of scientists who published over 30 papers in their first 10 or 20 career years. The error bars in this figure represent standard deviations. (c) Distributions of the number of communities (for $y = 30$) for scientists who started their career between 1950 and 1960, and for those who started their career between 1970 and 1980. The p-value of the Kolmogorov-Smirnov test is 0.098.(d) Distributions of the switching probability (for $y = 30$) of scientists who started their career between 1950 and 1960, and of those who started their career between 1970 and 1980. The p-value of the Kolmogorov-Smirnov test is 0.034.

Supplementary table

Supplementary Table 1. Kolmogorov-Smirnov test of the distributions of scientists' community number as well as the distributions of scientists' switching probability in different years.

p-value of Kolmogorov-Smirnov test on community number					p-value of Kolmogorov-Smirnov test on switching probability				
year	1940-1950	1950-1960	1960-1970	1970-1980	year	1940-1950	1950-1960	1960-1970	1970-1980
1940-1950	-	0.8114	0.9999	0.9307	1940-1950	-	0.0381	$1.80{*}10^{-3}$	$1.11{*}10^{-7}$
1950-1960		-	0.3458	0.2897	1950-1960		-	$4.77{*}10^{-4}$	$1.64{*}10^{-11}$
1960-1970			-	0.5967	1960-1970			-	$9.87{*}10^{-6}$
1970-1980				-	1970-1980				-

We consider the scientists in APS data who started their careers in each adjacent ten years, e.g. 1940-1950, 1950-1960, 1960-1970, 1970-1980. (left) p-value of the Kolmogorov-Smirnov test of the distribution of scientists' number of communities in different year periods. (right) p-value of the Kolmogorov-Smirnov test of the distribution of scientists' switching probability in different year periods. The p-values are all larger than 0.2 when comparing the distribution of number of communities in different year periods, supporting the assumption that these data follow similar distributions. However, the p-values are all smaller than 0.04 when comparing the distribution of scientists' switching probability in different year periods, suggesting significant differences between these distributions.

---

[1] Sinatra, R., Deville, P., Szell, M., Wang, D. & Barabasi, A.-L. A centuary of physics, *Nat. Phys.* **11**, 791-796 (2015).

[2] Blondel, V. D., Guillaume, J.-L., Lambiotte, R. & Lefebvre, E. Fast unfolding of communities in large networks, *J. Stat. Mech.* P10008 (2008).

[3] Balassa, B. Trade liberalization and "revealed" comparative advantage. *Manchester School* 33, 99-123 (1965).

[4] Shen, H.-W. & Barabasi, A.-L. Collective credit allocation in science, *Proc. Natl. Acad. Sci. USA* **111**, 12325-12330 (2014).

[5] Reichardt, J. & Bornholdt, S. Statistical Mechanics of Community Detection, *Phys. Rev. E* **74**, 016110 (2006).

[6] Rosvall, M. & Bergstrom, C. T. Maps of random walks on complex networks reveal community structure, *Proc. Natl. Acad. Sci. USA* **105**, 1118-1123 (2008).

[7] Kawamoto, T. & Rosvall, M. Estimating the resolution limit of the map equation in community detection, *Phys. Rev. E* **91**, 012809 (2015).
