# Supplementary material for: Increasing trend of scientists to switch between topics
Source: Nat Commun. 2019 Jul 31;10:3439. doi: 10.1038/s41467-019-11401-8 (PMC6668429; doi:10.1038/s41467-019-11401-8)
Supplement: Supplementary file 3 — Reporting Summary [file 41467_2019_11401_MOESM3_ESM.pdf]

natureresearch

Corresponding author(s): Shlomo Havlin

Last updated by author(s): Jun 23, 2019

Reporting Summary

Nature Research wishes to improve the reproducibility of the work that we publish. This form provides structure for consistency and transparency in reporting. For further information on Nature Research policies, see Authors & Referees and the Editorial Policy Checklist .

Statistics

For all statistical analyses, confirm that the following items are present in the figure legend, table legend, main text, or Methods section.

n/a	Confirmed	
☐	☒	The exact sample size (n) for each experimental group/condition, given as a discrete number and unit of measurement
☐	☒	A statement on whether measurements were taken from distinct samples or whether the same sample was measured repeatedly
☐	☒	The statistical test(s) used AND whether they are one- or two-sided<br>*Only common tests should be described solely by name; describe more complex techniques in the Methods section.*
☐	☒	A description of all covariates tested
☐	☒	A description of any assumptions or corrections, such as tests of normality and adjustment for multiple comparisons
☐	☒	A full description of the statistical parameters including central tendency (e.g. means) or other basic estimates (e.g. regression coefficient) AND variation (e.g. standard deviation) or associated estimates of uncertainty (e.g. confidence intervals)
☐	☒	For null hypothesis testing, the test statistic (e.g. F, t, r) with confidence intervals, effect sizes, degrees of freedom and P value noted<br>*Give P values as exact values whenever suitable.*
☒	☐	For Bayesian analysis, information on the choice of priors and Markov chain Monte Carlo settings
☒	☐	For hierarchical and complex designs, identification of the appropriate level for tests and full reporting of outcomes
☐	☒	Estimates of effect sizes (e.g. Cohen's d, Pearson's r), indicating how they were calculated

Our web collection on statistics for biologists contains articles on many of the points above.

Software and code

Policy information about availability of computer code

Data collection	The data used in this paper are all publicly accessible.
Data analysis	The community structure of the network is detected with the fast unfolding algorithm which is a heuristic method based on modularity optimization [J. Stat. Mech. P10008 (2008)].

For manuscripts utilizing custom algorithms or software that are central to the research but not yet described in published literature, software must be made available to editors/reviewers. We strongly encourage code deposition in a community repository (e.g. GitHub). See the Nature Research guidelines for submitting code & software for further information.

Data

Policy information about availability of data

All manuscripts must include a data availability statement. This statement should provide the following information, where applicable:

- Accession codes, unique identifiers, or web links for publicly available datasets
- A list of figures that have associated raw data
- A description of any restrictions on data availability

The data used in this paper are all publicly accessible. The American Physical Society data can be downloaded via https://journals.aps.org/datasets, and the computer science data can be downloaded via https://www.aminer.cn/aminernetwork.

Field-specific reporting

Please select the one below that is the best fit for your research. If you are not sure, read the appropriate sections before making your selection.

Behavioural & social sciences study design

All studies must disclose on these points even when the disclosure is negative.

Study description	We study the research dynamics of scientists by constructing a network of each individual scientist's publications characterizing their co-citing relations.
Research sample	We analyze the publication data from all journals of American Physical Society (APS). Another set of data that we analyzed in the supplementary materials is the computer science data obtained by extracting scientists' profiles from on-line Web databases.
Sampling strategy	As the network size needs to be large enough to ensure meaningful community detection results, we consider in this study all scientists that have published more than 20 (or 50) papers in the data set. In APS data, we found and analyzed 3,420 authors with at least 50 papers, and 15,373 authors with at least 20 papers. In computer science data, we found and analyzed 9,818 authors in this data with at least 50 papers.
Data collection	The data used in this paper are all publicly accessible. The American Physical Society data can be downloaded via https://journals.aps.org/datasets, and the computer science data can be downloaded via https://www.aminer.cn/aminernetwork.
Timing	The American Physical Society data ranges from year 1893 to year 2010. The computer science data ranges from year 1948 to year 2014.
Data exclusions	No data were excluded from the analysis.
Non-participation	No dropout participates.
Randomization	Participates were not allocated into experimental groups.

Reporting for specific materials, systems and methods

We require information from authors about some types of materials, experimental systems and methods used in many studies. Here, indicate whether each material, system or method listed is relevant to your study. If you are not sure if a list item applies to your research, read the appropriate section before selecting a response.

Materials & experimental systems

n/a	Involved in the study	
☐	☐	Antibodies
☐	☐	Eukaryotic cell lines
☐	☐	Palaeontology
☐	☐	Animals and other organisms
☐	☐	Human research participants
☐	☐	Clinical data

Methods

n/a	Involved in the study	
☐	☐	ChIP-seq
☐	☐	Flow cytometry
☐	☐	MRI-based neuroimaging

Antibodies

Antibodies used	*Describe all antibodies used in the study; as applicable, provide supplier name, catalog number, clone name, and lot number.*
Validation	*Describe the validation of each primary antibody for the species and application, noting any validation statements on the manufacturer's website, relevant citations, antibody profiles in online databases, or data provided in the manuscript.*

Eukaryotic cell lines

Policy information about cell lines

Cell line source(s)	*State the source of each cell line used.*
Authentication	*Describe the authentication procedures for each cell line used OR declare that none of the cell lines used were authenticated.*
Mycoplasma contamination	*Confirm that all cell lines tested negative for mycoplasma contamination OR describe the results of the testing for mycoplasma contamination OR declare that the cell lines were not tested for mycoplasma contamination.*
Commonly misidentified lines (See ICLAC register)	*Name any commonly misidentified cell lines used in the study and provide a rationale for their use.*
